# Adaptive Neuro-Fuzzy Fusion of Multi-Sensor Data for Monitoring a Pilot’s Workload Condition

**DOI:** 10.3390/s19163629

**Published:** 2019-08-20

**Authors:** Xia Zhang, Youchao Sun, Zhifan Qiu, Junping Bao, Yanjun Zhang

**Affiliations:** 1 College of Civil Aviation, Nanjing University of Aeronautics & Astronautics, Nanjing 211106, China; 2 Shanghai Aircraft Design & Research Institute, Commercial Aircraft Corporation of China, Ltd., Shanghai 201210, China; 3 School of Mechanical Engineering, Yangzhou University, Yangzhou 225127, China

**Keywords:** aircraft pilot, workload, multi-source data fusion, fuzzy neural network, principal component analysis, parameter learning

## Abstract

To realize an early warning of unbalanced workload in the aircraft cockpit, it is required to monitor the pilot’s real-time workload condition. For the purpose of building the mapping relationship from physiological and flight data to workload, a multi-source data fusion model is proposed based on a fuzzy neural network, mainly structured using a principal components extraction layer, fuzzification layer, fuzzy rules matching layer, and normalization layer. Aiming at the high coupling characteristic variables contributing to workload, principal component analysis reconstructs the feature data by reducing its dimension. Considering the uncertainty for a single variable to reflect overall workload, a fuzzy membership function and fuzzy control rules are defined to abstract the inference process. An error feedforward algorithm based on gradient descent is utilized for parameter learning. Convergence speed and accuracy can be adjusted by controlling the gradient descent rate and error tolerance threshold. Combined with takeoff and initial climbing tasks of a Boeing 737–800 aircraft, crucial performance indicators—including pitch angle, heading, and airspeed—as well as physiological indicators—including electrocardiogram (ECG), respiration, and eye movements—were featured. The mapping relationship between multi-source data and the comprehensive workload level synthesized using the NASA task load index was established. Experimental results revealed that the predicted workload corresponding to different flight phases and difficulty levels showed clear distinctions, thereby proving the validity of data fusion.

## 1. Introduction

The rapid development of the air transportation industry puts forward higher requirements on aircraft performance and safety than ever before. By means of structural optimization, system integration, and redundant design, airborne systems are being highly complicated and coupled. However, the cockpit is the only interface between pilot and aircraft, integrating all the human–machine interaction equipment required to perform flight tasks. Novel features constantly arise during pilot–aircraft interaction, including large-scale information, simplex interactive mode, multiple interactive nodes, and high real-time requirements. Therefore, it is highly possible for pilots to be caught in an unbalanced workload condition. Future advanced flight support systems are expected to monitor and evaluate the pilot’s real-time workload with multi-source data, which could obtain a timely and effective warning of overload status. The same holds true for ground controllers of unmanned aerial vehicles (UAV), in which case, workload still cannot be neglected, although the pilot is detached from the aircraft. Furthermore, it is of great benefit for improving the design of human–machine interaction and flight operation procedures to ensure flight safety.

Related research in pilot workload domains mainly covers two directions. Among them, aviation physiologists and psychologists concentrate on the mechanism of workload initiated by cognition, while human factors engineers hold interests in qualitative and quantitative evaluation of human–machine interaction design. The productions of these studies include, but are not limited to, the changing rule of physiological characteristic parameters or statistical subjective feedbacks of operators.

In the 1960s and 1980s, aerospace research institutes gradually developed evaluation techniques like Cooper–Harper scales, NASA task load index (NASA-TLX) and subjective workload assessment technique (SWAT) [1,2,3,4], and successfully applied them in various types of aircrafts. However, such methods mostly rely on pilot’s subjective feedback and thus there exist limitations in reflecting workload comprehensively. Later work tended to combine physiological characteristics with evaluation scales to increase the credibility of conclusions. Nocera et al. analyzed the sensitivity of the spatial dispersion index to mental workload changes based on a pilot’s eye movements in different flight phases [5]. Borghini et al. quantified the relationship between neurophysiological activities and mental workload [6]. Hoepf et al. addressed the feasibility of using physiological data to evaluate cognitive workload [7]. Then, they compared the advantages and disadvantages of different neural network models in assessment, and found that the evaluation results of different models were not always consistent with subjective perceptions, which should be strengthened in terms of adaptability [8]. Based on NASA-TLX and Likert scales, Orlandi et al. studied the effects of shiphandling maneuvers on psychological and physiological reactions of marine pilots in combination with heart rate, heart rate variability, and pupil dilation [9]. Wulvik et al. found the relationship between mental state and physiology in a simulated control room, but it lacked the quantitative description of mathematical models [10]. Ziegler et al. compared workload-based performance in two simulated flight tasks to determine different sensors’ predictive power, but the work was limited to only the direct comparison of physiological indexes [11]. A general concern about the aforementioned studies is the generalization ability of the workload model. The physiological parameters that could best reflect the workload remains controversial. As a result, the major limitation appears when we have to give up the existing one to rebuild another from scratch if other physiological indices are added into the model.

Other research has focused on the practicability of evaluation techniques, and thus were centered on improving subjective assessment methods. Liu et al. proposed to investigate the effects of time pressure and target uncertainty on a UAV operator’s workload based on subjective scores and secondary task performance [12]. Rusnock et al. reported on a new method called workload profiles to continuously evaluate workload without interrupting operations, avoiding the widely known inability of subjective evaluation [13]. Chi et al. used subjective mental workload ratings, adapted from NASA-TLX scales, to help determine the training time for new workers to get familiar with new tasks [14]. Nevertheless, the main weakness still lies in how to handle the widely varied personal opinions to reach a convincing conclusion.

Even though there is an persistent call for the intelligent identification of an operator’s workload, it has only been partly implemented in some fields, including automatic driving vehicle, artificial intelligence, and medical science. 

Kong et al. presented a driving fatigue recognition method based on the phase lock value (PLV) of electroencephalogram (EEG) signal [15]. In addition, they also developed a driving fatigue detection system based on machine vision and an AdaBoost algorithm by capturing the driver’s real-time facial and eyeball features [16]. Sun et al. proposed an adaptive dynamic recognition model for vehicle driver’s fatigue based on a multi-level feature fusion [17]. Through introducing dynamic basic probability assignment (BPA), the weight of each feature source can be adapted to the change of fatigue. Ke et al. constructed a mental workload recognition model using feature selection and regression analysis of an EEG [18]. What makes their work stand out is the supportability for cross-task situations. Zammouri et al. designed an EEG workload classifier combining power spectral density (PSD) analysis and statistical criteria. Based on this, a brain–computer interface was developed for real-time workload evaluation in cognitive learning [19]. McDonald et al. proposed machine learning approaches for detecting driver distraction, with a training set of 21 algorithms [20].

What can be summarized from above-mentioned studies is that physiological parameter recording and analysis is still one of the most popular measures in workload detection. It has advantages in real-time performance while other means fail. In the domain of physiology and medicine, research used physiological indices to monitor health condition as well. Yamada et al. studied the method of identifying mental fatigue in different age groups based on eye movement indicators [21]. Lamti et al. established a mental workload assessment model by fusing the features in an EEG [22]. Zhang et al. fused EEG spectral and temporal features using two-stream neural networks for mental workload assessment [23].

An operator’s workload condition is dually affected by his physiological state and task performance in a complex task environment. As a result, research emphasis should be placed on the strong coupling between the two factors. For monitoring the pilot’s workload condition in the cockpit, both real-time performance and generalization ability of the proposed method should receive significant attention. In contrast to the aforementioned methods, the model proposed in this paper aimed to assess a pilot’s real-time workload condition quantitatively by fusing multi-source physiological and flight data based on a fuzzy neural network. More specifically, to cover the shortage of qualitative and delayed evaluation, this study raises an objective solution by mapping real-time captured multi-source data to an overall workload level. Additionally, to make up the deficiency of incompatibility and one-sidedness from model to model, this study leaves the expansibility for any data flows concerned with workload to future research.

The remainder of this paper is divided into five sections. Section 2 gives a detailed explanation for each part of the model. Section 3 addresses the parameter learning method to help realize the ability of self-adaption. Section 4 outlines a case to demonstrate the feasibility and reasonability of applying the data fusion model to monitor the pilot’s real-time workload. Section 5 discusses the experimental results from different perspectives. Finally, conclusions are drawn in Section 6.

## 2. Model Structure

In a real flight environment, pilots need to make cognitive decisions in constantly changing situations according to their own abilities acquired from training and work experience. Due to the uncertainty in probability, it is suitable to describe their behavior patterns with fuzzy system. However, owing to the lack of ability toward autonomous learning and adaption, modeling with a fuzzy system is clearly not commensurate with the intelligent attributes of pilots who act as agents in abstract models. On the other hand, a neural network has excellent learning performance by mining feature information from samples and adapting parameter values to the needs of different occasions, though it cannot form abstract rules to express knowledge. As a result, fuzzy neural network has been widely used in modeling nonlinear dynamic systems with the advantages of both sides. Based on this, a high-performance data fusion model can be constructed with feature parameters. For basic concepts about fuzzy neural networks, see References [24,25]. Meanwhile, refer to References [26,27,28,29] for performances of fuzzy neural networks in related studies such as flight control and fault diagnosis. Furthermore, they have also gained popularity in other areas of science such as pattern recognition, data classification, image processing, and robot control [30,31,32,33,34].

A pilot’s workload condition can be reflected by physiological responses during flight and the ability to maneuver the aircraft according to a flight plan. Therefore, the monitoring data related to the workload condition includes flight data and a pilot’s physiological data. Flight data can be obtained from sensors installed in the airframe structure or airborne system and saved in flight recorders. Key flight characteristic parameters include pitch angle, yaw angle, roll angle, airspeed, climbing rate, etc. Furthermore, the pilot’s physiological data can be collected from EEG, ECG, eye tracker, and the like. Key physiological characteristic parameters include heart rate, heart rate variability, respiratory rate, respiratory depth, pupil diameter, etc. Taking key flight or physiological characteristic parameters *x*_1_, *x*_2_, …, *x_k_* as inputs, and the pilot’s integrated workload level *y* as the output, the mapping between inputs and output can be expressed as:

(1) $$y=f\left(x_{1},x_{2},\cdots ,x_{k}\right)$$

**Figure 1:**
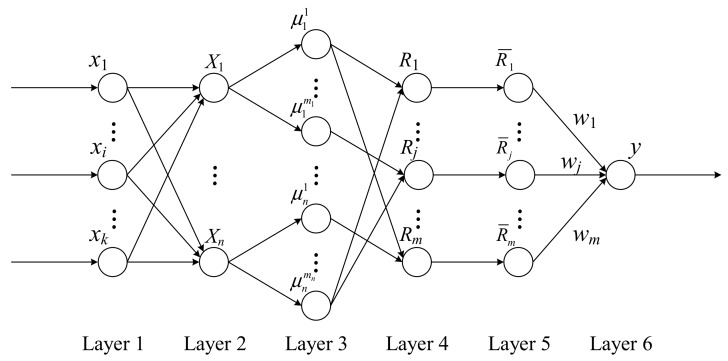
Fuzzy neural network model with a multi-layer structure.

Based on this mapping relationship, a fuzzy neural network model with multiple layers can be constructed, as shown in Figure 1.

In Layer 1, feature quantities *x_i_* (*i* = 1, 2, …, *k*) are extracted from original monitoring data as the inputs of the fuzzy neural network. The balance between accuracy and resource demand can be achieved via fusion with feature data rather than raw data. 

Layer 2 is the principal component extraction layer. The principal components of the input feature data are extracted through a linear transformation into a new variable *X_p_* (*p* = 1, 2, …, *n*). It can help guarantee the accuracy of results and further improve the fusion efficiency. 

Layer 3 is the fuzzification layer, determining the degree of membership of each feature quantity to different fuzzy attributes (*p* = 1, 2, …, *n*, *q* = 1, 2, …, *m_p_*). 

Layer 4 is the fuzzy rule-matching layer, calculating the applicability *R_j_* (*j* = 1, 2, …, *m*) of different fuzzy rules. 

Layer 5 is the normalization layer, which aims to normalize the applicability of different fuzzy rules. 

Layer 6 is the output layer. In this layer, the data fusion result will be defuzzified using connection weight *w_j_* (*j* = 1, 2, …, *m*), and the pilot’s integrated workload level is the output.

Overall, the model conforms to the basic structure of Mamdani fuzzy neural networks [35]. What makes it stand out from standard models is the dimension reduction process before fuzzification. Thus, it is expected to improve fusion efficiency when dealing with complex flight tasks compared with other similar applications.

### 2.1. Principal Component Analysis

High coupling exists in the feature data that is inputted into the model. Flight characteristic parameters, such as pitch angle, yaw angle, roll angle, airspeed, and climbing rate, are interrelated with each other under the constraints of flight dynamics. Pilot’s physiological characteristic parameters, such as heart rate, heart rate variability, respiratory rate, respiratory depth, pupil diameter, etc., are linked as the task scenario changes. In order to reduce the complexity, the main information of the feature data is extracted using principal component analysis. Thereby, the original *k* variables can be linearly combined to form *n* new variables, and then a fuzzy membership relationship will be constructed for the reasoning analysis.

Assume that the observation matrix of *k* variables in a monitoring time series [1,t] is:

(2) $$X={\left[\begin{array}{cccc} x_{11} & x_{12} & \cdots & x_{1k}\\ x_{21} & x_{22} & \cdots & x_{2k}\\ \vdots & \vdots & \ddots & \vdots \\ x_{t1} & x_{t2} & \cdots & x_{tk} \end{array}\right]}_{t\times k}$$

After normalization, the correlation coefficient matrix of *X* is

(3) $$R={\left[\begin{array}{cccc} r_{11} & r_{12} & \cdots & r_{1k}\\ r_{21} & r_{22} & \cdots & r_{2k}\\ \vdots & \vdots & \ddots & \vdots \\ r_{k1} & r_{k2} & \cdots & r_{kk} \end{array}\right]}_{k\times k}$$

where *r_ij_* represents the covariance of *x_i_* and *x_j_* (*i*, *j* = 1, 2, …, *k*).

Based on the eigenvalues *λ_i_* (*i* = 1, 2, …, *k*) of matrix *R*, the respective contribution rates of different principal components can be calculated using:

(4) $$C_{i}=\frac{\lambda_{i}}{\sum_{i=1}^{k}\lambda_{i}},\;\;i=1,2,\cdots ,k$$

According to the cumulative contribution rate of principal components, the number of new combination variables *X_p_* (*p* = 1, 2, …, *n*) can be determined. It is generally considered that if the cumulative contribution rate reaches 70–90%, combination variables can represent most information of the original variables [36].

Taking the eigenvector *a_i_* = [*a_i_*_1_ *a_i_*_2_ … *a_ik_*] (*i* = 1, 2, …, *k*) of matrix *R* to be linear transform coefficients, the original variables *x_i_* (*i* = 1,2, …, *k*) can be combined into *n* new ones using:

(5) $$X_{p}=a_{p1}x_{1}+a_{p2}x_{2}+\cdots +a_{pk}x_{k},\;p=1,2,\cdots ,n$$

By using new combination variables for fuzzy reasoning, the scale of fuzzy layer nodes will be compressed to $1/\prod_{p=n+1}^{k}m_{p}$ of the original one. Therefore, the efficiency of the data fusion can be improved.

### 2.2. Fuzzy Membership

Layer 3 of the fuzzy neural network is used to determine the membership of different combination variables to each fuzzy attribute; that is to say, the input characteristic quantities are blurred in this layer.

In order to maintain the balance between control precision and operation speed, applicable fuzzy partitions should be determined after several repeated attempts. If eight fuzzy partitions are chosen, for example, then *m*_1_ = *m*_2_ = … = *m_n_* = 8 in this layer. The corresponding fuzzy language values are NB (negative big), NM (negative medium), NS (negative small), NZ (negative zero), PZ (positive zero), PS (positive small), PM (positive medium), and PB (positive big).

In order to obtain better control sensitivity, the membership function can be a composite form of Z-shaped, Gaussian, and S-shaped membership functions, namely:

- Z-shaped membership function:
  (6) $$\mu_{p}^{q}=\left\{\begin{array}{cc} 1, & X_{p}\leq a_{pq}^{Z}\\ 1-2{\left(\frac{X_{p}-a_{pq}^{Z}}{b_{pq}^{Z}-a_{pq}^{Z}}\right)}^{2}, & a_{pq}^{Z}\leq X_{p}\leq \frac{a_{pq}^{Z}+b_{pq}^{Z}}{2}\\ 2{\left(\frac{X_{p}-a_{pq}^{Z}}{b_{pq}^{Z}-a_{pq}^{Z}}\right)}^{2}, & \frac{a_{pq}^{Z}+b_{pq}^{Z}}{2}\leq X_{p}\leq b_{pq}^{Z}\\ 0, & X_{p}\geq b_{pq}^{Z} \end{array}\right.$$
  where $a_{pq}^{Z}$ denotes the shoulder value and $b_{pq}^{Z}$ denotes the foot value of the Z-shaped membership function.
- Gaussian membership function:
  (7) $$\mu_{p}^{q}=e^{-\frac{{\left(x-c_{pq}\right)}^{2}}{2\sigma_{pq}^{2}}}$$
  where *c_pq_* denotes the mean value and *σ_pq_* denotes the standard deviation of the Gaussian membership function.
- S-shaped membership function:
  (8) $$\mu_{p}^{q}=\left\{\begin{array}{cc} 0, & X_{p}\leq a_{pq}^{S}\\ 2{\left(\frac{X_{p}-a_{pq}^{S}}{b_{pq}^{S}-a_{pq}^{S}}\right)}^{2}, & a_{pq}^{S}\leq X_{p}\leq \frac{a_{pq}^{S}+b_{pq}^{S}}{2}\\ 1-2{\left(\frac{X_{p}-a_{pq}^{S}}{b_{pq}^{S}-a_{pq}^{S}}\right)}^{2}, & \frac{a_{pq}^{S}+b_{pq}^{S}}{2}\leq X_{p}\leq b_{pq}^{S}\\ 1, & X_{p}\geq b_{pq}^{S} \end{array}\right.$$
  where $a_{pq}^{S}$ denotes the foot value and $b_{pq}^{S}$ denotes the shoulder value of the S-shaped membership function.

**Figure 2:**
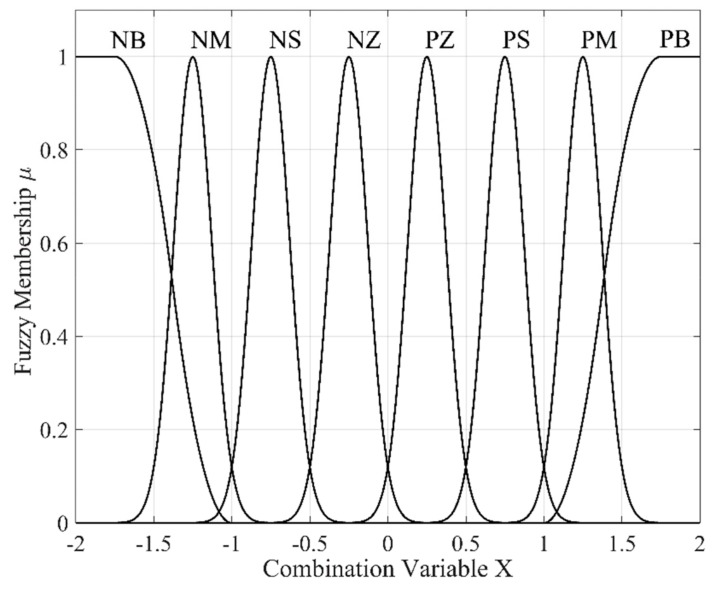
Composite membership function graph.

The composite membership function graph, with initial parameters $a_{pq}^{Z}=-1.75$, $b_{pq}^{Z}=-1$, $c_{pq}=0.1213$, $\sigma_{pq}\epsilon \left\{\pm 1.25,\pm 0.75,\pm 0.25\right\}$, $a_{pq}^{S}=1$, and $b_{pq}^{S}=1.75$, is shown in Figure 2.

### 2.3. Fuzzy Control Rules

Each node in Layer 4 represents a fuzzy rule. Under the premise that the number of new combination variables *X_p_* in Layer 2 is *n*, the number of nodes in Layer 4, denoting *m*, can be determined using:

(9) $$m=\overset{n}{\underset{p=1}{\prod }}m_{p}$$

According to the logical relationship between the inputs and outputs of the fuzzy neural network, fuzzy control rules can be constructed based on “if-then” language, which is like:

If (*X*_1_ is NB) and (*X*_2_ is NB) and … and (*X_n_* is NB), then (*y* is NB).

If (*X*_1_ is NM) and (*X*_2_ is NB) and … and (*X_n_* is NB), then (*y* is NB).

…

If (*X*_1_ is PB) and (*X*_2_ is PB) and … and (*X_n_* is PB), then (*y* is PB).

Obviously, in real conditions, the fuzzy membership of each fuzzy control rule is not consistent. Define the applicability of fuzzy control rule, *R_j_* (*j* = 1, 2, …, *m*), to represent the minimum value of membership function in antecedent rules according to:

(10) $$R_{j}=\min\left\{\mu_{1}^{q_{1}},\mu_{2}^{q_{2}},\cdots ,\mu_{n}^{q_{n}}\right\}$$

where *q_p_* = 1, 2, …, 8 (*p* = 1, 2, …, *n*).

After determining the applicability of each fuzzy control rule, in order to ensure the comparability of a pilot’s integrated workload level and speed up the optimization algorithm for parameter learning based on a gradient descent, Layer 5 normalizes the applicability by calculating:

(11) $${\bar{R}}_{j}=\frac{R_{j}}{\sum_{j=1}^{m}R_{j}},\;j=1,2,\cdots ,m$$

### 2.4. Defuzzification

What the fuzzy neural network finally outputs in Layer 6 is the deblurred pilot’s integrated workload level by giving a connection weight *w_j_* (*j* = 1, 2, …, *m*) to each normalized applicability value in Layer 5. The calculation method can be expressed as:

(12) $$y=\left[\begin{array}{cccc} {\bar{R}}_{1} & {\bar{R}}_{2} & \cdots & {\bar{R}}_{m} \end{array}\right]\left[\begin{array}{c} w_{1}\\ w_{2}\\ \vdots \\ w_{m} \end{array}\right]={\bar{R}}_{1}\cdot w_{1}+{\bar{R}}_{2}\cdot w_{2}+{\bar{R}}_{m}\cdot w_{m}$$

If the fuzzy neural network needs to achieve multi-dimensional outputs, that is, to construct a multi-input and multi-output (MIMO) mapping relationship, then the defuzzification calculation method can be expressed as:

(13) $$\left[\begin{array}{c} y_{1}\\ y_{2}\\ \vdots \\ y_{r} \end{array}\right]=\left[\begin{array}{cccc} w_{11} & w_{12} & \cdots & w_{1m}\\ w_{21} & w_{22} & \cdots & w_{2m}\\ \vdots & \vdots & \ddots & \vdots \\ w_{r1} & w_{r2} & \cdots & w_{rm} \end{array}\right]\left[\begin{array}{c} {\bar{R}}_{1}\\ {\bar{R}}_{2}\\ \vdots \\ {\bar{R}}_{m} \end{array}\right]$$

where *r* indicates the number of outputs.

## 3. Parameter Learning

**Figure 3:**
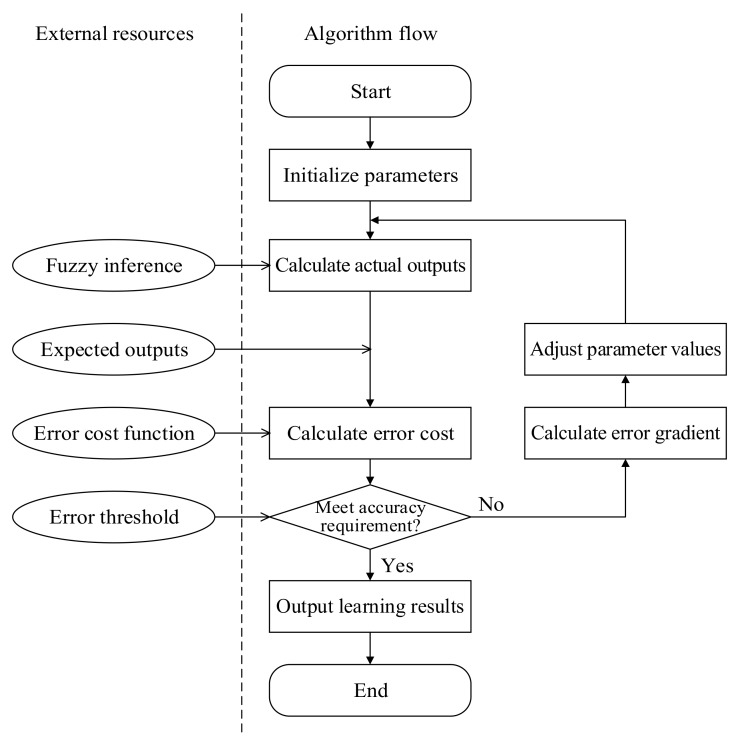
Flow chart of error feedforward algorithm based on gradient descent.

The weights connecting Layers 5 and 6, namely *w_j_*, need to be determined through adaptive learning. An error feedforward algorithm based on a gradient descent can be applied for parameter learning, and the algorithm flow is shown in Figure 3.

For a fuzzy neural network with *r*-dimensional outputs, the error cost function is:

(14) $$Ec=\overset{r}{\underset{i=1}{\sum }}{\left(y_{ei}-y_{ai}\right)}^{2}/2$$

where *y_ei_* represents the *i*th expected output and *y_ai_* represents the *i*th actual output.

The first-order gradient of the error cost function in the direction of the weight variable is:

(15) $$\frac{\partial Ec}{\partial w_{ij}}=\frac{\partial Ec}{\partial y_{i}}\cdot \frac{\partial y_{i}}{\partial w_{ij}}=-\left(y_{ei}-y_{ai}\right){\bar{R}}_{j}$$

where *w_ij_* denotes the weight connecting the *i*th node in Layer 6 and *j*th node in Layer 5; ${\bar{R}}_{j}$ denotes the value of the *j*th node in Layer 5.

The iterative equation for error feedforward parameter learning based on a gradient descent is:

(16) $$w_{ij}\left(k+1\right)=w_{ij}\left(k\right)-\beta \frac{\partial Ec}{\partial w_{ij}},\;\;i=1,2,\cdots ,r,\;\;j=1,2,\cdots ,m$$

where *β* represents the parameter learning rate. The convergence rate of parameter learning can be controlled by adjusting the gradient falling rate.

Substituting Equation (15) into Equation (16), the cumulative calculation equation can be derived to be:

(17) $$w_{ij}=\overset{r}{\underset{i=1}{\sum }}\overset{m}{\underset{j=1}{\sum }}\beta {\bar{R}}_{j}\left(y_{ei}-y_{ai}\right)$$

By setting an error tolerance threshold *τ*, the iterative learning process will be terminated when suitable parameter values are obtained. At this point, the fuzzy neural network will reach the optimal state via training.

The formalized description of the algorithm is shown in Table 1.

## 4. Case Study

**Figure 4:**
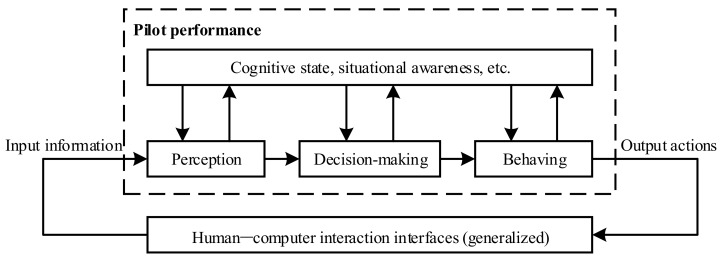
Human–machine–environment closed-loop circuit in the cockpit.

From the perspective of cognitive psychology, individual performance can be measured from multiple aspects including: (1) time to perform the task, (2) accuracy in performing the task, and (3) neurological data [37]. In the cockpit, the “human–machine–environment” closed-loop circuit during a flight mission is shown in Figure 4. A pilot’s integrated workload level can be reflected by the physiological state, time requirement, and task performance. These elements run through the overall process composed of perception, decision-making, behaving, etc.

In this section, a typical flight task from a civil aircraft operation manual will be taken as background. By constructing the “human-in-the-loop” simulation flight scenario, physiological parameter-measuring instruments, flight-parameter recorder, and other sensing devices were used to capture the state characteristics of the flight simulator and personnel. A multi-sensor data fusion model was constructed based on a fuzzy neural network to realize the mapping from a multi-source feature data to workload level. Through these studies, we wish to offer an effective solution for monitoring the pilot’s workload condition in real time.

### 4.1. Synthetic Flight Simulation Experimental System

**Figure 5:**
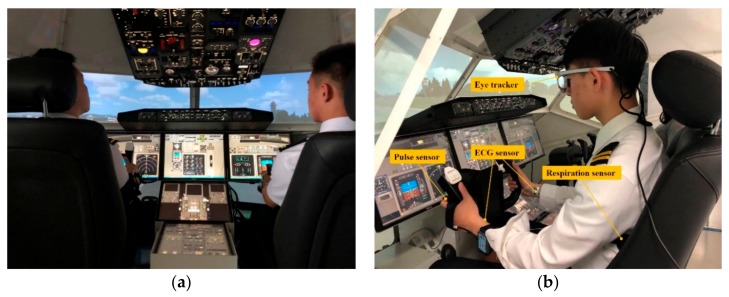
Experiment scenes of flight simulation. (**a**) Flight simulation experimental platform. (**b**) Flight personnel wearing physiological monitoring sensors during the experiment.

The synthetic flight simulation experimental system is mainly composed of a Boeing 737–800 cockpit simulator, annular vision module, physiological monitoring module, flight data recording module, and general control station. The cockpit simulator and annular vision provide an immersive operation experience for flight personnel. A physiological monitoring module obtains real-time physiological characteristics through an SMI eye tracker (SensoMotoric Instruments company, Teltow, Germany) and a BIOPAC multi-channel physiological recorder (BIOPAC Systems Inc., Goleta, USA). The flight data recording module transmits the time-varying data of dynamic parameters during flight manipulation to the general control station. The experiment scenes are shown in Figure 5.

### 4.2. Simulated Flight Scene

We referred to the Shanghai Airlines Standard Operating Procedures (SOP) for a Boeing 737–800 aircraft, and supposed the left-seat pilot maneuvers the aircraft. It was also assumed that the takeoff course was in the same direction as the runway. Analog experimental operation procedures were developed on the basis of a normal takeoff and initial climbing procedures. The steps of responding to the air traffic controller (ATC) commands, setting the heading, setting the navigation mode, etc., which were not closely related to the direct flight maneuver, were omitted to form a simplified version, as shown in Table 2.

Since the left-seat pilot was responsible for maneuvering the aircraft, the evaluator performed the role of a right-seat pilot in the simulated flight in order to make the variables of the left-seat pilot relatively independent. Regardless of the interactions between pilots due to task assignments, it was assumed that the tasks of the right-seat pilot can be performed accurately and in a timely manner.

According to the setting of the simulated flight scene, key indicators for measuring the pilot’s workload condition extracted from flight data during takeoff and the initial climbing phase included pitch angle, heading, and airspeed [38,39]. Key indicators that characterize the state of the pilot extracted from physiological data included heart rate, heart rate variability, respiratory rate, respiratory depth, pupil diameter, gaze time, glance time, and blink time.

### 4.3. Feature Extraction

Left-seat flight personnel were composed of fourteen people who already had training experience in similar flight simulators such as Boeing 737 series, Airbus 320 series, and Cessna 172. Before the formal experiment, they had mastered the flight control and operation procedures of a Boeing 737–800 simulator through necessary instruction and training.

In the experiment, each person encountered three different abnormal conditions, including primary flight display (PFD) images disappearing, left aileron jamming, and left engine in-flight shutdown. Any failure was randomly triggered during the takeoff and the initial climbing phase so that the flight personnel could not make predictions in advance. Therefore, no psychological expectation would be considered either.

Flight and physiological data under different conditions were recorded in real time. Once finishing each flight mission, the flight personnel filled in NASA-TLX scale questionnaires according to their own experience. Subjective evaluations of flight utility were carried out based on six dimensions: mental demand (MD), physical demand (PD), temporal demand (TD), performance (OP), effort (EF), and frustration (FR) [2,3]. 

In different simulated flight scenes, three kinds of commands, including start and stop commands, flight control commands, and fault observation commands, were extracted as segment signs for different flight phases, namely:

- Start and stop commands: “experiment starts,” “experiment ends.”
- Flight control commands: PF calling “take off,” PM calling “130 knots,” PM calling “rotate,” PF calling “raise the landing gear,” PF calling “flaps up,” PM calling “flare out.”
- Fault observation commands: “PFD images disappear,” “left engine stops.”

Among them, PFD images disappearing and left engine in-flight shutdown belong to explicit failures, that is, PF/PM can obtain fault information through display interfaces. The difference is that the former can be obtained directly by PF through the visual channel, but the latter was acquired by the PF capturing PM’s calling through the auditory channel. In addition, left aileron jamming was set to be an implicit failure, that is, PF/PM could not obtain relevant information through any display interface. The purpose was to distinguish PF’s physiological characteristics under unknown failure conditions.

These commands were helpful to segment each flight process into different phases including taxiing, normal climbing, maneuvering under failure, and flaring out.

According to the requirements of different single-point failures or combined failures on PF’s flight maneuvering skill, the mission difficulty can be divided into three levels:

- Low level (LL): Only PFD images disappeared.
- Medium level (ML): Both PFD images disappearing and left aileron jamming occurred.
- High level (HL): Both PFD images disappearing and left engine in-flight shutdown occurred.

The most influential factors for subjective evaluation after the flight are failure conditions in flight and PF’s efforts to maintain the desired maneuvering target after the failure. Data characteristics of PF encountering different failures were extracted from multi-source data streams. The nonlinear mapping relationship between multi-source data features and NASA-TLX multi-dimensional subjective workload evaluation was then established.

Feature data after encountering different failures in 42 sets of experiments are shown in Table 3, Table 4 and Table 5. Subjective workload evaluation results using NASA-TLX are shown in Table 6.

### 4.4. Multi-Source Feature Fusion

Input parameters included pitch angle (*x*_1_), heading (*x*_2_), airspeed (*x*_3_), heart rate (*x*_4_), heart rate variability (*x*_5_), respiratory rate (*x*_6_), respiratory depth (*x*_7_), pupil diameter (*x*_8_), gaze time (*x*_9_), glance time (*x*_10_), and blink time (*x*_11_). A multi-source data fusion model based on a fuzzy neural network can be built to characterize the pilot’s workload condition with NASA-TLX’s six-dimensional indicators.

The flight and physiological characteristic data initially input into the model have defects such as non-uniform dimensions and inconsistent time-varying regularity. It is easy to cause low computational efficiency and data logic confusion via direct fusion. Therefore, it is necessary to standardize and squeeze the original feature data first.

#### 4.4.1. Data Standardization

What can be obtained from flight characteristic data is the pilot’s ability to stably maintain the current flight control state after the failure. In this paper, the degree of stability was measured using population standard deviations of pitch angle (*x*_1_), heading (*x*_2_), and airspeed (*x*_3_) during the takeoff and initial climbing phase.

What can be obtained from the physiological characteristic data is the pilot’s physiological and psychological stress when they try to maintain the established flight plan after the failure. In this paper, the degree of concentration and tension was measured using mean values of heart rate (*x*_4_), heart rate variability (*x*_5_), respiratory rate (*x*_6_), respiratory depth (*x*_7_), and pupil diameter (*x*_8_), as well as statistical proportions of gaze time (*x*_9_), glance time (*x*_10_), and blink time (*x*_11_). 

Based on the research results in the fields of physiology, medicine, ergonomics, etc. [40,41,42,43], the above-mentioned physiological characteristic parameters can be divided into the following two categories according to the change relations with workload. The first category contains the parameters positively related to workload level, namely heart rate (*x*_4_), heart rate variability (*x*_5_), respiratory rate (*x*_6_), pupil diameter (*x*_8_), and gaze time (*x*_9_). The second category contains the parameters negatively related to workload level, namely respiratory depth (*x*_7_), glance time (*x*_10_), and blink time (*x*_11_).

Considering the individual differences in flight and physiological characteristic data, the maximum and minimum values of characteristic parameters remain unknown. In a few cases, outlier data beyond the normal range of values will also appear. Therefore, the z-score standardization method was adopted for data preprocessing, transforming original data to standard normal distributed sequences using:

(18) $$y_{i}=\frac{x_{i}-\bar{x}}{s},\;\;i=1,2,\cdots ,n$$

where $\bar{x}=\frac{1}{n}\sum_{i=1}^{n}x_{i}$ calculates the mean value and $s=\sqrt{\frac{1}{n-1}\sum_{i=1}^{n}{\left(x_{i}-\bar{x}\right)}^{2}}$ calculates the population standard deviation of the original data.

For the subjective evaluation results after the flight, since the NASA-TLX scale already limits the score range to [0,100], the output data did not need to be standardized. However, in order to achieve a concentrated opinion score, the six-dimensional NASA-TLX scores should be averaged on the assumption that each factor holds a comparable weight in this given task.

#### 4.4.2. Dimension Reduction

In order to improve the efficiency of data fusion, principal component analysis was then performed on the standardized feature data to reduce the data size in the fuzzification layer. During the flight process, characteristic parameters have a strong coupling, with the change of one parameter causing changes through the linkage with others. After the dimension reduction process, the contribution rate of each principal component and the cumulative contribution rate of the first *n* (*n* = 1, 2, …, 11) principal components are shown in Table 7.

It can be seen from Table 6 that the cumulative contribution rate of the first four and five principal components reached 77.16% and 85.44%, respectively, which can meet the standard set in Jolliffe [36] to be able to cover most of the original information. Using the eigenvectors of the correlation coefficient matrix as coefficients to linearly combine the normalized physiological characteristic data in Table 2, Table 3 and Table 4, expressions of the first five principal components are listed below:

(19) $$\begin{array}{ll} P_{1}=0.1158x_{1} & +0.7878x_{2}+0.1080x_{3}+0.0173x_{4}+0.0777x_{5}-0.0481x_{6}-0.0258x_{7}\\ \; & -0.3583x_{8}-0.3346x_{9}+0.3219x_{10}+0.0320x_{11} \end{array}$$

(20) $$\begin{array}{ll} P_{2}=0.0715x_{1} & -0.3914x_{2}+0.1602x_{3}-0.0265x_{4}+0.0031x_{5}+0.2395x_{6}-0.3314x_{7}\\ \; & -0.1859x_{8}-0.4805x_{9}+0.1213x_{10}+0.6066x_{11} \end{array}$$

(21) $$\begin{array}{ll} P_{3}=-0.1712x_{1} & -0.3945x_{2}-0.2053x_{3}+0.0625x_{4}-0.0845x_{5}-0.3474x_{6}+0.4606x_{7}\\ \; & -0.3897x_{8}-0.2858x_{9}+0.3883x_{10}-0.2117x_{11} \end{array}$$

(22) $$\begin{array}{ll} P_{4}=-0.1228x_{1} & -0.1576x_{2}-0.0230x_{3}-0.1472x_{4}+0.6068x_{5}-0.0859x_{6}-0.5377x_{7}\\ \; & -0.2883x_{8}-0.0039x_{9}-0.0388x_{10}-0.4342x_{11} \end{array}$$

(23) $$\begin{array}{ll} P_{5}=-0.0400x_{1} & -0.0149x_{2}-0.3290x_{3}+0.0966x_{4}-0.3903x_{5}+0.5371x_{6}-0.3160x_{7}\\ \; & +0.1071x_{8}-0.0225x_{9}+0.4379x_{10}-0.3691x_{11} \end{array}$$

#### 4.4.3. Data Fusion

The number of variables in Layer 2, *n*, was set be four or five for data fusion. By weighing the fusion accuracy and computing cost, the number could be finally determined.

By selecting the suitable fuzzy membership and fuzzy control rules through several attempts, the fuzzy neural network can be used to train the connection weight *w_j_* between Layer 5 and Layer 6. In this case, fuzzy membership functions were chosen to be shaped like those in Section 2.2, but the number of fuzzy partitions was set to be four, with initial parameters $a_{pq}^{Z}=-1.5$, $b_{pq}^{Z}=-0.5$, $c_{pq}=0.2690$, $\sigma_{pq}\epsilon \left\{\pm 0.5\right\}$, $a_{pq}^{S}=0.5$, and $b_{pq}^{S}=1.5$. The number of *w_j_* appeared as an exponent relation to the number of variables in Layer 2. The changes in scale can also reflect the consumption of computing resources. Different parameter learning rates cause different convergence speeds. When the number of principal components is large, choosing a high learning rate can speed up the process of data fusion.

1. *n* = 4

**Figure 6:**
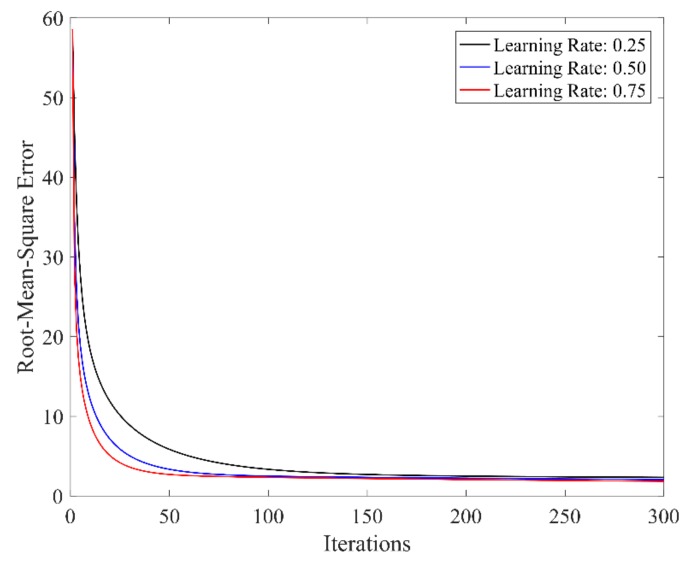
Relationship between error cost and learning times under different learning rates (*n* = 4).

**Figure 7:**
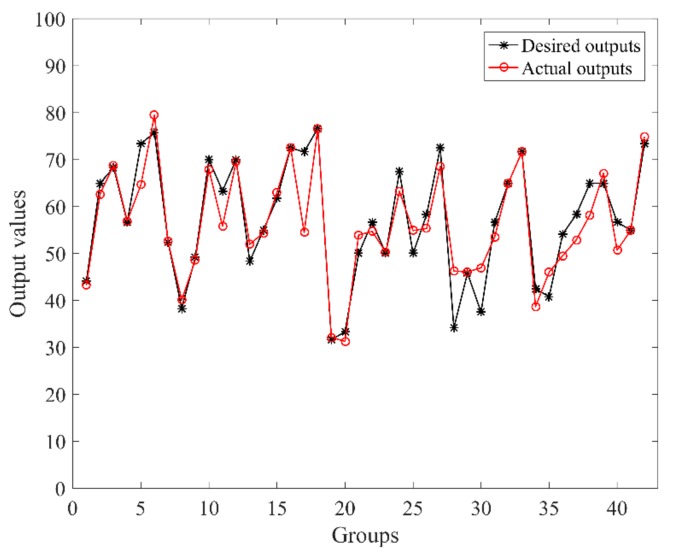
Comparison between desired outputs and actual outputs (*β* = 0.75, RMSE = 4.85).

In this case, the number of *w_j_* was 256. Due to the fact that the minimum division value of the NASA-TLX scale is 5, the error threshold was also set to be 5. When choosing the parameter learning rate *β* to be 0.25, 0.50, and 0.75, the convergence relation between the output error cost and learning times is shown in Figure 6. Under the learning rate of 0.75 and error tolerance threshold of 5, the final error cost was 4.85 after 21 learning iterations. The comparison between the desired and actual outputs is shown in Figure 7.

2. *n* = 5

**Figure 8:**
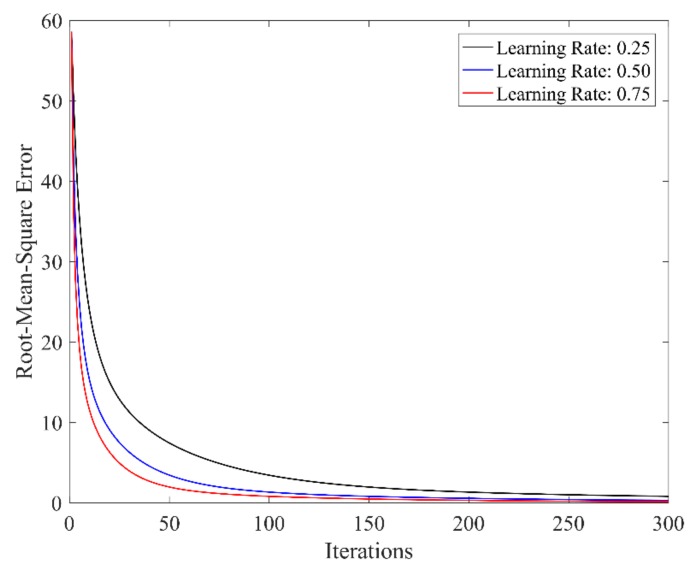
Relationship between error cost and learning times under different learning rates (n = 5).

**Figure 9:**
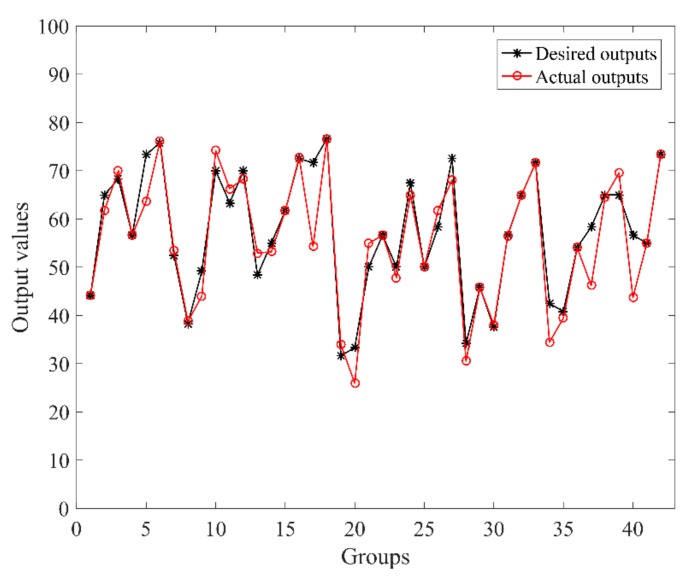
Comparison between desired outputs and actual outputs (β = 0.75, RMSE = 4.96).

In this case, the number of *w_j_* is 625. With parameter learning rate 0.25, 0.50, and 0.75 respectively, the convergence relation between output error cost and learning times is shown in Figure 8. Under the learning rate 0.75 and error tolerance threshold 5, the final error cost is 4.96 after 25 times’ learning. The comparison between desired and actual outputs is shown in Figure 9. 

Comparison from the perspectives of parameter learning rate, number of iterations, and root-mean-square error under different numbers of variables in Layer 2 is shown in Table 8.

It can be seen that as the number of variables increased, the number of iterations within the expected error threshold range increased significantly, and the computing cost increased gradually. In addition, the root-mean-square error increased overall, such that the ratio of benefit to cost continued to deteriorate. It can be concluded that, although increasing the number of principal components could improve the convergence precision, it was at the cost of computing resources. When the error cost first fell into the predetermined threshold, both the iteration speed and error values in the first case (*n* = 4) are superior to those in the second case (*n* = 5). As a result, the first four principal components of flight and physiological characteristic data were extracted for modeling.

## 5. Discussion

By comparing the same flight personnel’s workload during different flight phases in the same simulated flight, or the workload when encountering different failures in different simulated flights, the model performance of feature data fusion and workload prediction could be verified. The prediction results are shown in Table 9.

Consistent with the NASA-TLX scale, the predicted workload values in different flight phases are shown in Table 10.

There were four abnormal values that exceeded the normal value range [0,100] in prediction results, accounting for 2.38% of the total number. The main reasons for this phenomenon may include:

- Abnormal feature attributes in sampling data before fusion:

This may be caused by environmental changes or interference from other channels during sensor measurement. The model could not map these data points into a normal value range according to normal decision logic.

- Side effects of using the z-score normalization method in data preprocessing:

On the one hand, the z-score standardization method is applicable to the case where the maximum or minimum value is uncertain, which is consistent with the characteristics of individual differences in physiological characteristic parameters. On the other hand, the z-score normalization method cannot eliminate abnormal data outside the normal range, while it also avoids the impact of abnormal points on the overall data set.

- Limitations of the training sample sets:

The fuzzy neural network was trained based on the data samples during failure. The data samples collected in this stage contained obvious feature attributes, which had advantages in identifying or predicting the severe workload. However, it showed insufficient sensitivity for flight phases where feature changes were not obvious and overall workload stayed low.

**Figure 10:**
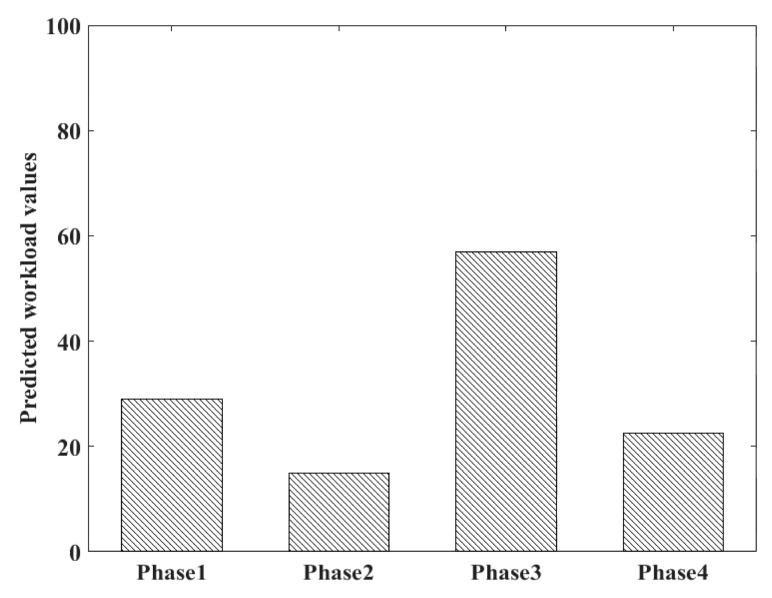
Mean predicted workload values during different flight phases (Phase 1: Taxiing, Phase 2: Normal Climbing, Phase 3: Maneuvering Under Fault, & Phase 4: Flaring Out).

In this study, training samples obtained during failure could also form the baseline where workload stayed at a high level. The establishment of a baseline is one of the most common handling methods in workload research [44,45]. Using this method, data samples collected in other phases can function as validation sets to evaluate the performance. Through contrasting statistical workload values in different flight phases or at difficulty levels, determining whether the proposed model serves the purpose of distinguishing the changing workload reasonably can be done. The predicted workload values in taxiing, normal climbing, maneuvering under failure, and flaring out are shown in Figure 10. Obviously, the mean workload under failure conditions was the highest, and workload in the taxiing phase was higher than that in the normal climbing phase. The reason may be complex. When the aircraft is taxiing, flight personnel need to observe different information such as alignment with the runway, takeoff speed, and rotation speed. In this case, the workload is mainly composed of mental load, while the pilot only needs to control the attitude angle in a normal climbing stage, with physical load playing a leading role. Furthermore, workload in a flaring out stage fell between the ones in the taxiing phase and normal climbing phase. It can be seen that the impact of failures was greater than that of operation complexity on maneuvering performance.

**Figure 11:**
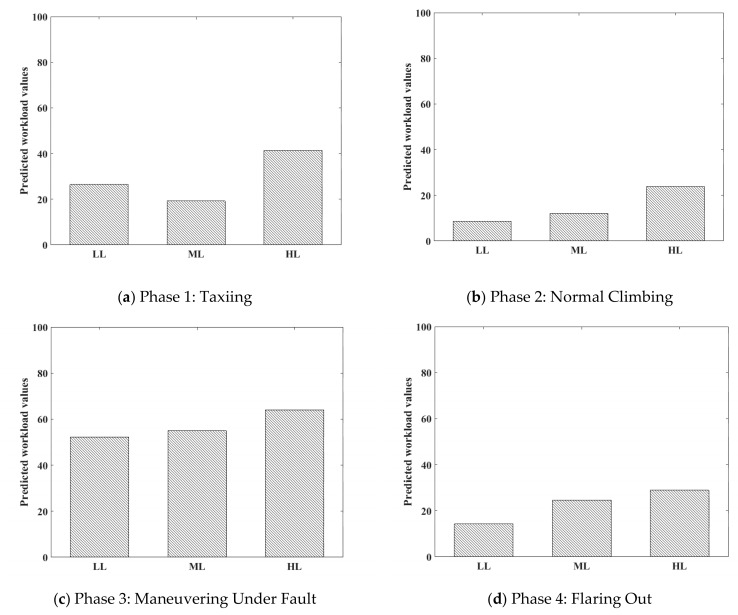
Mean predicted workload values under different mission difficulties.

In different flight phases, the average workload under different difficulty levels predicted by the model is shown in Figure 11. It can be seen that workload values could not distinguish between each other before the single-point failures or combined failures were triggered (Phases 1 & 2). After the failure was triggered, the workload increased significantly with the increase of operation difficulty, and Phases 3 and 4 exhibited the same phenomenon. Obviously, the effect of “left engine in-flight shutdown” was greater than that of “left aileron jamming” on flight maneuvering. Because of the existence of standby instruments, “PFD images disappearing” had the least impact on flight maneuvering, which is consistent with the pre-experimental assumption and basic perception of flight.

To further our research, more physiological measuring means should be applied to monitor the pilot’s workload condition. For instance, another feature source we have decided to take into account is EEG data, which is well acknowledged for its close connection with mental workload. However, due to the proficiency requirements of data collection and analysis, it remains controversial regarding how to quantify the relations between brain waves and mental workload. Despite that, we hope to enhance the fusion ability of our model on accuracy, sensitivity, and robustness with the introduction of such physiological indexes. The validity of this method can be further proved using questionnaire surveys among test pilots or airline pilots. To apply workload-monitoring techniques in real flights, other issues to consider mainly include the impacts on flight safety and compatibility with airborne systems. In other words, whether real-time workload monitoring will meet airworthiness requirements still requires much exploration.

## 6. Conclusions

In this paper, a multi-source data fusion model for monitoring the pilot’s workload condition based on a fuzzy neural network is proposed. The case analysis of simulated takeoff and initial climbing process shows that the model can realize the fusion of key flight and physiological characteristic parameters such as pitch angle, heading, airspeed, heart rate, heart rate variability, respiratory rate, respiratory depth, pupil diameter, fixation time, glance time, and blink time. It also possesses the ability to map to an integrated workload level characterized by NASA-TLX’s six-dimensional metrics. Furthermore, the fusion and prediction results could distinguish the different flight phases and maneuvering difficulties based on multi-source feature data. 

The proposed model reveals the mechanism of mapping physiological and flight feature data to overall workload. It gives insight into the cross-coupled factors contributing to the pilot’s mental state and makes the most of multi-source information to avoid one-sidedness. Furthermore, it realizes the transformation from experience-dependent estimation to a data-driven evaluation in the field of man–machine system science. Moreover, in real-world applications, what are studied in this paper can provide basic support for real-time monitoring and early warning of the pilot’s workload status. Specifically, in the stage of airworthiness verification, the work can help to recognize the factors that may result in overload and improving the design of interfaces or operating procedures. In the stage of pilot training, the findings can also serve as the evaluation basis of training effects. In the stage of airline operation, the monitoring technique can warn crew members of an overload state to prevent further deterioration, thereby reducing the risk of air crew disability.

## Figures and Tables

**Table 1:** Formalized description of the error feedforward algorithm.

**Error Feedforward Algorithm for Parameter Learning**	
**Inputs:**	initialized weights, *w_j_* (*j* = 1, 2, …, *m*);
	learning rate *β*;
	error tolerance threshold *τ*.
**Process:**	calculate initial actual outputs with fuzzy inference;
	calculate initial error cost with error cost function (14);
	**while** error cost falls outside the threshold **do**
	calculate error gradient with Equation (15);
	update weight values with Equation (17);
	update actual outputs;
	update error cost;
	**end while**
**Outputs:**	updated weights, *w_j_* (*j* = 1, 2, …, *m*).

**Table 2:** Analog takeoff and initial climbing operation procedures.

Step No.	Pilot Flying (PF)	Pilot Monitoring (PM)
1	Align the runway, and be ready to take off when airspeed is greater than 45 knots.	Slowly push the thrust handle and slide out the aircraft when N1 is within 40%.
2	Call “take off” when airspeed is greater than 45 knots.	Push thrust to 40% N1, and make the left and right thrusts symmetrical. Call “40, stable” when parameters are stable.
3	Push the steering column a little forward and keep the direction with the rudder.	Call “130 knots” when the airspeed is 3–5 knots in advance of 130 knots.
4	Answer “130 knots” and hold the steering column to get ready to rotate.	Call “rotate” when airspeed is 140 knots.
5	Softly rotate (2°–3° per second) at *V_R_* speed. Then, bring the aircraft to an initial attitude angle of 15°.	Call “positive climbing rate” when the altimeter indicates positive climbing rate.
6	Call “raise the landing gear” after confirming positive climbing.	Raise the landing gear when hearing the calling.
7	Hold the flight attitude and rise at a speed of 165–175 knots.	Call “1000 feet” when reaching the height of 1000 feet.
8	Call “flaps up” when observing the speed is above 165 knots and there is a clear growth trend.	Place the flap handle at 0, and answer “flaps back in place” when the flaps indicate 0.
9	-	Call “3000 feet” at 3000 feet.
10	Call “vertical navigation.”	Push the thrust to 80%.
11	-	Call “10,000 feet, flare out” at 10,000 feet.
12	Put the pitch angle to about 3° and keep the flight altitude constant.	Call “experiment ends” when flight height remains the same.

**Table 3:** Flight maneuvering data after encountering different failures.

No.	Pitch Angle (º)	Heading (º)	Airspeed (knot)
1	14.1804 ± 0.9652	19.0713 ± 2.0091	241.2167 ± 19.5601
2	14.5003 ± 0.9831	32.8499 ± 12.4234	217.8288 ± 5.9969
3	3.3836 ± 4.5923	203.5062 ± 104.9897	248.8197 ± 36.4469
4	9.7670 ± 3.1108	36.5237 ± 3.6697	315.9794 ± 22.2091
5	14.3072 ± 1.5077	45.3139 ± 4.9348	229.9576 ± 10.7507
6	9.4049 ± 0.3696	55.1342 ± 1.5970	240.4584 ± 2.2986
7	10.8644 ± 3.2643	2.1881 ± 1.0388	284.6951 ± 29.8569
8	13.7026 ± 3.1533	3.9796 ± 1.4385	237.5278 ± 5.9237
…	…	…	…
…	…	…	…
41	9.8007 ± 2.5607	17.2384 ± 3.5800	269.0684 ± 12.3520
42	10.6944 ± 2.3686	62.4950 ± 75.8252	212.1871 ± 22.8060

**Table 4:** ECG and respiratory data after encountering different failures.

No.	Heart Rate (bpm ^1^)	Heart Rate Variability (s)	Respiratory Rate (bpm ^2^)	Respiratory Depth (mm)
1	68.8887 ± 26.7930	1.0493 ± 0.5808	32.7495 ± 40.1275	0.0437 ± 0.0618
2	83.3899 ± 16.9637	0.7534 ± 0.1930	39.8135 ± 45.4972	0.0489 ± 0.0394
3	92.2354 ± 27.9626	0.6922 ± 0.1510	50.1296 ± 52.7483	0.0461 ± 0.0581
4	62.4001 ± 27.4923	1.3688 ± 1.2429	22.4469 ± 21.5953	0.0596 ± 0.0685
5	97.8012 ± 34.8229	0.6572 ± 0.1410	33.1376 ± 38.5239	0.0662 ± 0.0749
6	108.3679 ± 28.1629	0.5881 ± 0.1397	56.7218 ± 50.4941	0.1250 ± 0.1403
7	78.9282 ± 6.2099	0.7644 ± 0.0553	57.4063 ± 58.5690	0.0201 ± 0.0296
8	74.8417 ± 11.8903	0.8564 ± 0.3492	56.0719 ± 61.6657	0.0186 ± 0.0212
…	…	…	…	…
…	…	…	…	…
41	76.5368 ± 22.6353	0.9255 ± 0.5599	30.6861 ± 29.0981	0.0656 ± 0.0787
42	92.1149 ± 32.7781	1.1694 ± 2.9794	35.0873 ± 37.9755	0.0776 ± 0.0921

^1^ For heart rate, the unit bpm means the number of contractions (beats) of the heart per minute. ^2^ For respiratory rate, the unit bpm means breaths per minute.

**Table 5:** PFs’ raw eye movement data after encountering different failures.

No.	Pupil Diameter (mm)	Gaze Time Proportion (%)	Glance Time Proportion (%)	Blink Time Proportion (%)
1	4.4931 ± 0.5287	83.77	10.48	5.67
2	4.5686 ± 0.8766	74.39	12.92	12.17
3	4.5864 ± 0.5637	83.16	12.44	4.40
4	5.0246 ± 0.6515	76.06	16.06	7.83
5	4.6916 ± 0.5034	79.45	15.63	4.91
6	5.0036 ± 0.1890	75.45	24.55	0
7	4.2354 ± 0.7318	64.94	21.84	12.79
8	3.6905 ± 1.1745	44.86	26.42	28.01
…	…	…	…	…
…	…	…	…	…
41	0.8113 ± 0.8943	35.75	10.71	1.95
42	0.9810 ± 1.4161	18.70	31.84	0.12

**Table 6:** Subjective workload evaluation results using the NASA-TLX scale.

No.	MD	PD	TD	OP	EF	FR
1	65	70	60	10	50	10
2	80	95	65	30	90	30
3	80	90	65	50	90	35
4	80	75	50	25	70	40
5	80	90	70	70	80	50
6	80	90	70	65	80	70
7	85	70	55	25	50	30
8	25	50	60	30	35	30
…	…	…	…	…	…	…
…	…	…	…	…	…	…
41	70	75	50	30	60	45
42	85	90	70	50	85	60

**Table 7:** Principal component analysis of the feature data.

Principal Component No.	Contribution Rate (%)	Cumulative Contribution Rate (%)
1	29.48	29.48
2	19.93	49.41
3	17.75	67.16
4	10.00	77.16
5	8.28	85.44
6	5.10	90.54
7	4.32	94.86
8	2.98	97.84
9	1.66	99.50
10	0.36	99.86
11	0.14	100

**Table 8:** Data fusion results under different numbers of variables.

n	Learning Rate	Number of Iterations	Root-Mean-Square Error
4	0.25	61	4.94
	0.50	31	4.90
	0.75	21	4.85
5	0.25	74	4.86
	0.50	38	4.99
	0.75	25	4.96

**Table 9:** Workload prediction results in different flight phases.

No.	Taxiing	Normal Climbing	Maneuvering Under Fault	Flaring Out
1	40.68	3.49	44.12	0.00
2	18.30	18.87	64.73	6.06
3	60.69	0.00	68.65	37.92
4	14.63	2.69	56.69	7.21
5	13.55	12.66	72.74	0.50
6	44.23	57.39	75.95	102.26
7	21.15	4.00	52.51	−3.13
8	51.69	22.26	38.31	20.45
…	…	…	…	…
…	…	…	…	…
41	0	0.95	55.00	15.22
42	61.68	7.73	73.33	10.99

**Table 10:** Workload prediction results in different flight phases after unification with NASA-TLX.

No.	Taxiing	Normal Climbing	Maneuvering Under Fault	Flaring Out
1	40	5	45	0
2	20	20	65	5
3	60	0	70	40
4	15	5	55	5
5	15	15	75	0
6	45	55	75	100
7	20	5	55	**−5**
8	50	20	40	20
…	…	…	…	…
…	…	…	…	…
41	0	0	55	15
42	60	10	75	10
